# Age of menopause, healthy lifestyle and cardiovascular disease in women: a prospective cohort study

**DOI:** 10.1136/heartjnl-2024-324602

**Published:** 2024-12-17

**Authors:** Anushriya Pant, Alice A Gibson, Simone Marschner, Lee P Liao, Liliana Laranjo, Clara K Chow, Sarah Zaman

**Affiliations:** 1 Faculty of Medicine and Health, The University of Sydney Westmead Applied Research Centre, Westmead, New South Wales, Australia; 2 Menzies Centre for Health Policy and Economics, School of Public Health, Faculty of Medicine and Health, The University of Sydney, Sydney, New South Wales, Australia; 3 The University of Sydney Charles Perkins Centre, Sydney, New South Wales, Australia; 4 Department of Cardiology, Westmead Hospital, Westmead, New South Wales, Australia

**Keywords:** Cardiovascular Diseases, Cohort Studies, Risk Assessment, Risk Factors

## Abstract

**Background:**

Menopause is a timely opportunity to screen for cardiovascular disease (CVD) and intervene with healthier lifestyles. We investigated the association between premature/early menopause and the likelihood of CVD and whether a healthy lifestyle is associated with a lower likelihood of CVD in menopausal woman.

**Methods:**

The Sax Institute’s 45 and Up Study prospectively recruited participants aged ≥45 years (n=267 357) between 2005 and 2009 (New South Wales, Australia). Our study included women without prior CVD and reporting menopausal age at baseline. Primary outcome was new-onset CVD (self-reported heart disease/stroke) based on survey data at Wave 2 (2012–2015) and/or Wave 3 (2018–2020). Logistic regression models assessed the associations of premature (age <40 years) and early (age 40–44 years) menopause with CVD, compared with menopause between 50 and 52 years, adjusting for sociodemographic and clinical variables. Healthy lifestyle adherence was assessed using a score of five factors: smoking, physical activity, sitting, sleep and diet.

**Results:**

We included 46 238 women (mean age 62.1±8.2 years), with 5416 (11.7%) cases of CVD over 15-year follow-up. After adjustment, the odds of CVD was higher in women with premature menopause (OR 1.36, 95% CIs 1.17 to 1.59; p<0.0001) and early menopause (OR 1.15, 95% CI 1.03 to 1.28; p=0.013) compared with menopause between 50 and 52 years. Among all women, high (score 9–10) versus low (score 0–5) healthy lifestyle adherence led to 23% lower odds of CVD (OR 0.77, 95% CI 0.68 to 0.86; p<0.0001), and in women with premature menopause, led to 52% lower odds of CVD (OR 0.48, 95% CI 0.30 to 0.77, p=0.0022). Lifestyle effect did not significantly differ between menopause categories (interaction, p=0.71).

**Conclusion:**

Women with premature/early menopause are at higher likelihood for CVD. Lifestyle modification is associated with consistent reduction of the likelihood of CVD in women and should be encouraged across the life course.

**WHAT IS ALREADY KNOWN ON THIS TOPIC**

- While oestrogen provides cardioprotective effects in women, once menopause occurs, oestrogen decreases and the likelihood of cardiovascular disease (CVD) increases.
- The likelihood of CVD is higher in women who had menopause earlier; however, this information is not in current CVD risk assessment guidelines.

**WHAT THIS STUDY ADDS**

- Among an Australian cohort of women, menopause before age 45 was associated with elevated likelihood of CVD compared with menopause at between 50 and 52 years.
- Additionally, healthy lifestyle adherence, including five important factors of not smoking, sitting less, improving sleep, exercise and healthy diet, significantly reduced this likelihood of CVD, irrespective of age of menopause.

**HOW THIS STUDY MIGHT AFFECT RESEARCH, PRACTICE OR POLICY**

- Menopause is a timely opportunity to screen women, especially those with early menopause, for cardiovascular health and promote healthy lifestyle initiatives across the life course.

## Introduction

 Cardiovascular disease (CVD) is the leading cause of death globally and age of menopause onset significantly accelerates this likelihood among women.^1 2^ The past two decades has seen increased recognition of women’s CVD. However, historically, the under-representation of women has led to missed opportunities to prioritise CVD screening during menopause.^3^

The menopause stage is a critical window to intervene and potentially reduce a woman’s likelihood of developing CVD through lifestyle modification.^2^ This may involve targeting key cardiometabolic changes relevant in these women, especially those with earlier menopause.^2^ Contemporary literature demonstrates the link between adverse cardiovascular outcomes and earlier menopause,^4 5^ including the development of coronary heart disease (CHD),^6 7^ heart failure^8^ and premature mortality.^6 9^ Recently, in 2023, the updated Australian CVD risk guidelines recognised the menopause transition as an opportunity to screen for cardiovascular risk factors and CVD in women. However, female-specific risk factors like early menopause are not incorporated into the absolute CVD risk score. Additionally, there is limited data on the combined effect of lifestyle factors in mitigating the likelihood of CVD during and after menopause.^10 11^

We aimed to investigate the association between premature/early menopause and the likelihood of CVD in a population-based cohort of Australian women. We further aimed to investigate the effect of overall healthy lifestyle adherence on the likelihood of CVD in these women and whether the effect differed between menopause categories.

## Methods

### Study design and population

The Sax Institute’s 45 and Up Study is based in New South Wales (NSW), Australia. A total of 267 357 participants joined the 45 and Up Study between 2005 and 2009 by completing a baseline questionnaire (Wave 1) and giving signed consent for follow-up and linkage to health databases. Prospective participants were randomly selected from the Services Australia Medicare enrolment database, providing near complete population coverage. People aged 80+ years and rural/remote residents were oversampled. An estimated 19% of those invited participated and participants included ~11% of the NSW population aged ≥45 years. The first (Wave 2; 2012–2015) and second (Wave 3; 2018–2020) follow-up questionnaires were completed by 142 548/246 306 participants (response rate 58%) and 97 302/205 867 participants (response rate 47%), respectively.^12^ The study design and cohort profile have been fully described elsewhere.^13^

The current study used the baseline questionnaire to determine eligibility and menopause categories in female participants only. We included women reporting information on their menopause status and age of menopause. We excluded women with self-reported CVD at baseline and missing follow-up data for CVD. Women were categorised according to age of menopause as per standard definitions: premature menopause (before age 40 years), early menopause (between 40 and 44 years), relatively early menopause (between 45 and 49 years), median age of menopause at 50–52 years and late menopause (age >52 years). Menopausal categories were based on previous literature^4^ and commonly accepted guideline cut-offs.^2 14^

### Study outcomes

The primary outcome was self-reported CVD (defined as new-onset heart failure, stroke, and/or heart attack) based on two follow-up questionnaires: Wave 2 and Wave 3. Women were identified having first-onset CVD if they responded, ‘yes’ to any of the following: ‘Has a doctor ever told you that you have Heart Disease, Heart Failure, or Stroke?’, or ‘Has a doctor ever told you that you have Other Heart Disease?’ or ‘In the last month have you been treated for other Heart Disease?’ or ‘In the last month have you been treated for Heart Attack or Angina?’.

### Healthy lifestyle assessment

Lifestyle risk factors were identified from the baseline questionnaire. The full list of questions and response options is provided in online supplemental table S1. Lifestyle risk factors included smoking, physical activity, sleep duration, sitting time and diet. These were determined using the national guideline-specific advice for smoking, physical activity and diet.^15^ Recommendations for sleep and sitting were determined using previous studies.^15^,^17^ Participant responses to each lifestyle factor were categorised as low adherence, medium adherence and high adherence, with a score of 0, 1 and 2, respectively. Table 1 presents the distribution of lifestyle risk factors across the adherence levels. The combined healthy lifestyle score was calculated based on responses to each lifestyle risk factor, ranging from 0 to 10. A higher score indicated healthier lifestyle adherence. Participants were categorised into three adherence groups based on their combined healthy lifestyle score: high healthy lifestyle adherence (healthiest) (score 9–10), medium healthy lifestyle adherence (score 6–8) and low healthy lifestyle adherence (least healthy) (score 0–5). Low healthy lifestyle adherence was set as the reference group for all analyses. The conceptualisation and design of the combined lifestyle score has been published previously.^15^

**Table 1:** Distribution (%) of lifestyle risk factors across adherence levels in all women at baseline (n=46 238)

Lifestyle factor	Description	High adherence(score=2)	Medium adherence(score=1)	Low adherence(score=0)
Smoking	**Smoking history**	**Non-smoker**	**Previous smoker**	**Current smoker**
	N (%)	30 543 (66.06%)	13 295 (28.75%)	2399 (5.19%)
Physical activity	**MPVA minutes per week**	**≥300**	**150–299**	**≤149**
	N (%)	23 890 (55.23%)	7631 (17.65%)	11 731 (27.12%)
Sleep duration	**Hours per day**	**7–9**	**>5–7 or >9–11**	**<5 or >11**
	N (%)	36 364 (79.51%)	8409 (18.39%)	963 (2.11%)
Sitting	**Hours per day**	**<7**	**7–9**	**>9**
	N (%)	31 654 (72.19%)	7348 (16.76%)	4848 (11.06%)
Fruit	**Serves per day**	**≥2**	**1**	**0**
	N (%)	31 497 (68.78%)	12 057 (26.33%)	2238 (4.89%)
Vegetables	**Serves per day**	**≥5**	**3–4**	**0–2**
	N (%)	19 554 (42.39%)	16 133 (34.98%)	10 437 (22.64%)
Fish	**Serves per week**	**≥3**	**1–2**	**0**
	N (%)	10 270 (22.77%)	31 378 (69.58%)	3447 (7.64%)
Red meat	**Serves per week**	**0–2**	**3–4**	**≥5**
	N (%)	16 694 (36.42%)	20 186 (44.04%)	8952 (19.53%)
Processed meat	**Serves per week**	**0**	**1–2**	**≥3**
	N (%)	13 333 (33.00%)	22 312 (55.23%)	4752 (11.76%)
Diet score*	**Out of 10**	**>7**	**>3–7**	**0–3**
	N (%)	21 578 (46.78%)	22 541 (48.86%)	2012 (4.36%)
Combined healthy lifestyle score†	**Out of 10**	**9–10**	**6–8**	**0–5**
	N (%)	15 026 (33.04%)	26 233 (57.68%)	4222 (9.29%)

* Composite score based on response to the five diet questions.

† Composite score based on response to lifestyle factor questions.

MVPAmoderate to vigorous physical activity

### Confounders

Confounders were determined using the baseline questionnaire on self-reported sociodemographic variables, medical comorbidities and health status. Sociodemographic variables included age (continuous), ancestry (categorical: Australian, European, Asian, Other), highest qualification (categorical: no school certificate or other qualification, school or intermediate certificate, higher school to leaving certificate, trade or apprenticeship, certificate or diploma, and university degree or higher) and remoteness (categorical: using the Accessibility/Remoteness Index of Australia (ARIA+): metropolitan, inner regional, and outer regional/remote/very remote). Medical comorbidities and variables associated with health status included hypertension (categorical), diabetes mellitus (DM) (categorical), body mass index (BMI) (continuous), smoking status (categorical: current smoker, previous smoker and non-smoker) and menopausal hormone therapy (MHT) (formerly known as hormone replacement therapy) (categorical). All descriptions (questions and responses) and derivations for each variable are presented in online supplemental table S2.

### Statistical analysis

SAS V.9.4 for Windows was used to perform all analyses. Baseline characteristics were assessed across menopause categories. We used logistic regression analysis to calculate ORs and CIs for the prospective association between premature/early menopause and future likelihood of CVD. The following confounders were added to the adjusted model: age, BMI, ancestry, hypertension, DM, highest qualification, smoking status and MHT. Potential confounders were chosen using previous knowledge and univariate testing.^4 5^ We performed sensitivity analyses adjusting for other female-specific variables, including hypertension during pregnancy (HDP) (categorical), contraceptive pill (categorical), hysterectomy (categorical) and parity (continuous). These female-specific variables have been associated with attenuating effects on the likelihood of CVD.^18^,^20^ We also performed a sensitivity analysis adjusting for individual lifestyle behaviours (categorical) of physical activity, sleep duration, sitting time, and diet quality, and overall healthy lifestyle adherence.

For our secondary objective, we used logistic regression analysis to evaluate the association between overall healthy lifestyle adherence and the likelihood of CVD in this cohort of menopausal women, adjusting for age, BMI, ancestry, hypertension, DM, highest qualification, remoteness and MHT. We performed a sensitivity analysis with an additional adjustment for alcohol intake.

Interaction effects were analysed to determine if there is a modifying effect of healthy lifestyle adherence on the association of each menopause category with CVD outcome. We used a logistic regression model to assess the interaction of the overall healthy lifestyle adherence (categorical) with age of menopause (categorical) on the likelihood of the outcome CVD, adjusting for the following covariates of age, BMI, ancestry, hypertension, DM, highest qualification, remoteness and MHT.

We performed further sensitivity analyses to assess these associations with multiple imputation, imputing for missing covariate data. Multiple imputations for confounders with missing data were conducted using chained equations with the Markov chain Monte Carlo method, with the assumption of missing at random. We analysed women free from CVD in all analyses; all tests were two-sided and statistical significance was set at p≤0.05.

## Results

Of the 266 420 participants available for analysis, our final sample included 46 238 female participants who met eligibility (online supplemental figure S1). At baseline, women had a mean age 62.07±8.24 years, with a mean age at menopause of 49.44±5.10 years and mean BMI of 26.51±5.09 kg/m^2^. There were 1704 women with premature menopause (3.68%), 4297 women with early menopause (9.29%), 12 026 women with relatively early menopause (26.00%), 16 643 women with menopause at 50–52 years (35.99%) and 11 578 women with menopause after age 52 years (25.04%) (online supplemental table S3). Majority of women were from Australian/European ancestry (88.20%), lived in metropolitan NSW (50.27%), had university degrees/diplomas (50.67%) and were married/defacto (73.17%).

A higher proportion of women with premature menopause, compared with women with menopause at 50–52 years, had cardiovascular risk factors, including both traditional (eg, DM, hypertension, current/former smokers, being obese) and lifestyle (eg, physically inactive, poor sleep, unhealthy diet). These women were more likely to be on MHT, have hysterectomies and less likely to have used OCP.

### Association between menopause categories and the likelihood of CVD

After a 15-year follow-up, there were 5416 CVD events (11.7%). In the adjusted model, the odds of CVD was higher among those with premature menopause (OR 1.36, 95% CI 1.17 to 1.59; p<0.0001) and early menopause (OR 1.15, 95% CI 1.03 to 1.28; p=0.013) compared with those who had menopause between 50 and 52 years (table 2). There were no significant associations for the likelihood of CVD among women with relatively early (OR 1.07, 95% CI 0.99 to 1.16, p=0.094) or late menopause (OR 1.00, 95% CI 0.93 to 1.09, p=0.91).

**Table 2:** OR of cardiovascular disease according to menopause categories in women from the 45 and Up Study cohort (n=41 754)

	Menopause at 50–52 years	Premature menopause (<40 years)		Early menopause(40–44 years)		Relatively early menopause (45–49 years)		Late menopause(>52 years)	
	**Reference**	**OR**	**95% CI, p value**	**OR**	**95% CI, p value**	**OR**	**95% CI, p value**	**OR**	**95% CI, p value**
CVD									
Unadjusted	1.0 (reference)	1.43	(1.25 to 1.65), **p<0.0001**	1.13	(1.02 to 1.25), **p=0.017**	1.02	(0.94 to 1.09), p=0.68	1.06	(0.98 to 1.14), p=0.15
Adjusted*	1.0 (reference)	1.36	(1.17 to 1.59), **p<0.0001**	1.15	(1.03 to 1.28), **p=0.013**	1.07	(0.99 to 1.16), p=0.094	1.00	(0.93 to 1.09); p=0.91

Bolded p values are considered statistically significant at p<0.05.

All ORs and p values are in reference to mean age of menopause at 50–52 years.

* Adjusted for age, BMI, ancestry, baseline HTN, baseline DM, highest qualification, smoking status and *MHT*.

BMIbody mass indexCVDcardiovascular diseaseDMdiabetes mellitusHTNhypertensionMHTmenopausal hormone therapy

The effect sizes remained similar in all sensitivity analyses when adjusted for lifestyle behaviours (physical activity, sleep duration, sitting time and diet quality) and female-specific risk factors (past HDP, contraceptive pill, hysterectomies and parity) (online supplemental tables S4 and S5).

### Association between healthy lifestyle and the likelihood of CVD

We observed significant association for both medium and high healthy lifestyle adherence, compared with low healthy lifestyle adherence, on CVD after adjustment (table 3). High healthy lifestyle adherence was associated with lower odds of CVD compared with low healthy lifestyle adherence (OR 0.77, 95% CI 0.68 to 0.86, p<0.0001). Medium healthy lifestyle adherence was associated with lower odds of CVD compared with low healthy lifestyle adherence (OR 0.85, 95% CI 0.76 to 0.94, p=0.002). After further adjustments with alcohol intake, the effect sizes for high healthy lifestyle adherence (OR 0.76, 95% CI 0.68 to 0.85, p<0.0001) and medium healthy lifestyle adherence (OR 0.85, 95% CI 0.76 to 0.94, p=0.003) were similar to the main results (table 3).

**Table 3:** Association between cardiovascular disease and overall lifestyle adherence in all women from the 45 and Up Study cohort (n=41 672)

	Low healthy lifestyle adherence	Medium healthy lifestyle adherence		High healthy lifestyle adherence	
	**Reference**	**OR**	**95% CI, p value**	**OR**	**95% CI, p value**
CVD					
Unadjusted	1.0 (reference)	0.87	(0.79 to 0.96), **p=0.005**	0.78	(0.70 to 0.86), **p<0.0001**
Adjusted*	1.0 (reference)	0.85	(0.76 to 0.94), **p=0.002**	0.77	(0.68 to 0.86), **p<0.0001**
Additional adjustment for weekly alcohol intake	1.0 (reference)	0.85	(0.76 to 0.94), **p=0.003**	0.76	(0.68 to 0.85), **p<0.0001**

Bolded p values are considered statistically significant at p<0.05.

* Adjusted for age, BMI, ancestry, baseline HTN, baseline DM, highest qualification, remoteness and *MHT*.

BMIbody mass indexCVDcardiovascular diseaseDMdiabetes mellitusHTNhypertensionMHTmenopausal hormone therapy

**Figure 1:**
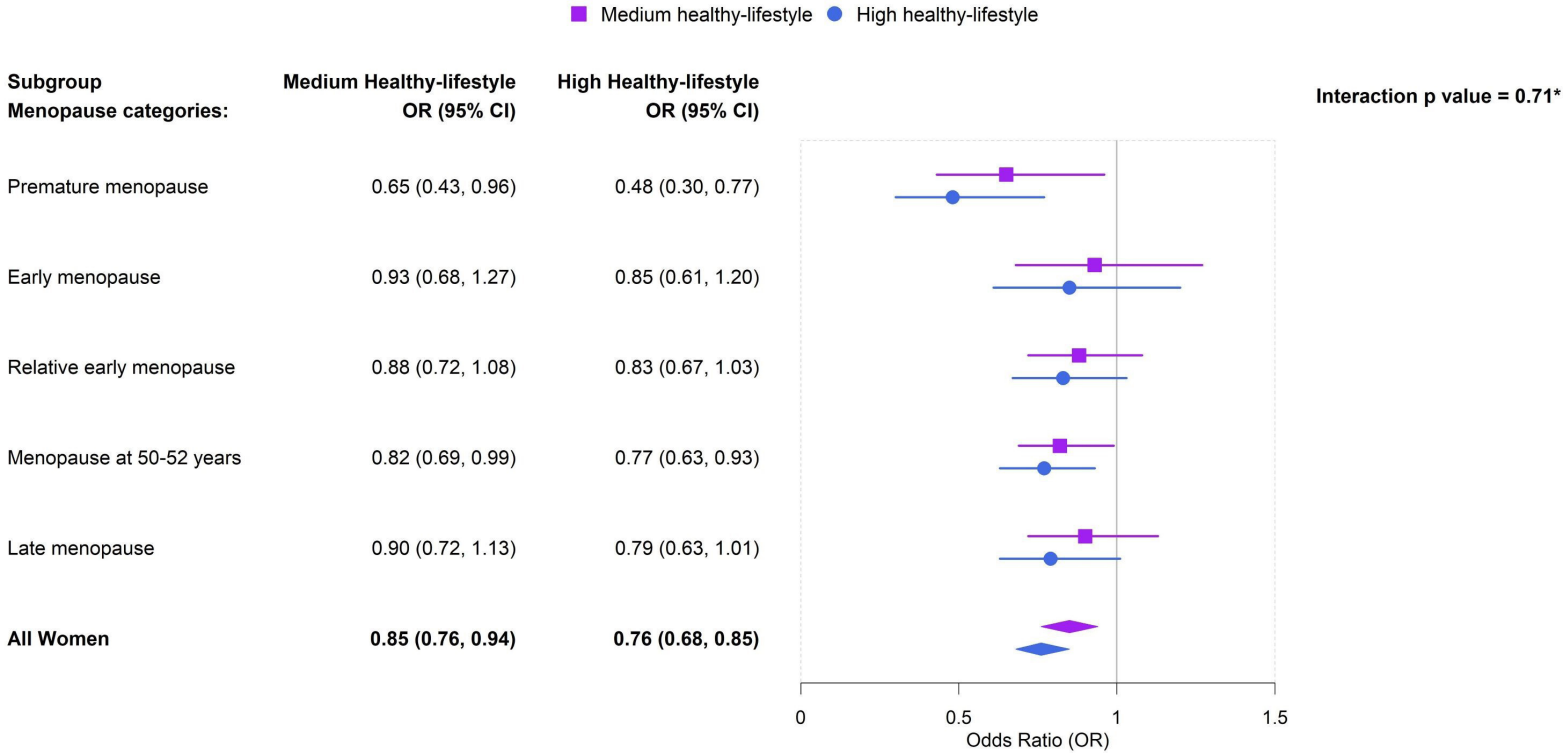
Forest plot of ORs for the association between overall healthy lifestyle adherence and the likelihood of cardiovascular disease (CVD) menopause categories in female participants from the 45 and Up Study cohort (N=46 238). Reference is low healthy lifestyle adherence. *Interaction effect between age of menopause and healthy lifestyle adherence on the likelihood of CVD was p=0.71.

We observed significant associations for both medium and high healthy lifestyle adherence, compared with low healthy lifestyle adherence, on CVD in only women with premature menopause and women with menopause between 50 and 52 years (figure 1). High versus low healthy lifestyle adherence was associated with lower odds of CVD among women with premature menopause (OR 0.48, 95% CI 0.30 to 0.77; p=0.002) and women with menopause between 50 and 52 years (OR 0.77, 95% CI 0.63 to 0.93, p=0.007) (online supplemental table S6). Medium versus low healthy lifestyle adherence was associated with lower odds of CVD among women with premature menopause (OR 0.65, 95% CI 0.43 to 0.96, p=0.030) and women with menopause between 50 and 52 years (OR 0.82, 95% CI 0.72 to 0.99, p=0.038) (online supplemental table S6).

On interaction analysis, there was no statistically significant interaction between age of menopause and healthy lifestyle score on the odds of CVD (p=0.71), as shown in figure 1. The lack of interaction indicated that the effect of overall healthy lifestyle did not differ by age of menopause on CVD and instead showed a consistent reduction of the likelihood of CVD in all menopause groups.

All sensitivity models imputing for missing values demonstrated consistent results (online supplemental tables S7–9).

## Discussion

Our study evaluated the prospective association between premature/early menopause and the likelihood of CVD in women aged ≥45 years who have reached menopause. To date, our study is the first Australian prospective study to assess the modifying effect of overall healthy lifestyle on CVD in population-based cohort of women who have reached menopause. Our healthy lifestyle score is unique with its composite score of five important risk factors including smoking, physical activity, sitting time, sleep duration and diet. We found that premature and early menopause were independently associated with 36% and 15% increased odds, respectively, for CVD over a 15-year follow-up period. High versus low adherence to a healthy lifestyle significantly reduced CVD odds in women by 23%.

Previous studies have indicated that earlier menopause is an important predictor for future cardiovascular events.^4 6^ In the current study, we included more than 45 000 women and found that premature menopause increased the likelihood of future CVD by 36%, while early menopause increased the likelihood of CVD by 15%. This was comparable to effect sizes in previous meta-analyses and prospective cohorts, ranging from 36% to 55% for premature menopause^4 21^ and from 19% to 50%^4 21^ for early menopause. These associations are consistent in different populations geographically, including East Asian,^4 21^ UK,^4^ US^4^ and European^4 21^ studies. Using data from the Multi-Ethnic Study of Atherosclerosis, Wellons *et al*^22^ found that early menopause was associated with a twofold increased risk for CHD, one of the highest effect sizes among longitudinal studies. However, this study was largely non-Caucasian, in contrast to our study (66.9% Caucasian/European), and other studies have indicated that age of menopause onset may differ by race/ethnicity.^23^ Most notably, in a meta-analysis of pooled individual studies from five regions (Australia, Scandinavia, USA, Japan and UK), Zhu *et al*^4^ found that every year of decrease in age of menopause was associated with a 3% increased risk of CVD. This study also demonstrated that additional adjustments for parity and contraceptive pill were consistent with the main results, similarly, shown in our sensitivity analyses that adjusted for these female-specific variables.

Endogenous oestrogen is well known for regulating various systemic pathways, including its protective effects on the vasculature, lipid metabolism and coagulation.^2^ The increased likelihood of CVD coincides with the reduction in oestrogen during menopause, which typically occurs between ages 44 and 55 years.^2^ Decreased levels of oestrogen have been linked with cardiometabolic alterations during menopause that may contribute to CVD development.^2^ First, menopause has been associated with weight gain and central adiposity, increasing the risk of obesity.^2^ Second, menopause is associated with changes to the lipid profile, regardless of ageing alone.^2^ Menopausal women often present higher levels of triglycerides and low-density lipoprotein-cholesterol and lower levels of high-density lipoprotein-cholesterol, consequently resulting in dyslipidaemia.^2 21^ Third, the prevalence of metabolic syndrome increases with menopause, possibly due to insulin resistance and impaired glucose metabolism, which in turn may lead to the development of type 2 DM (T2DM).^2 21^ Fourth, the reduction in oestrogen during menopause coincides with vascular changes.^2^ Oestrogen has vasodilatory effects by increasing the bioavailability of nitric oxide. However, decreased oestrogen in menopausal women might lead to vascular dysfunction and subsequent high blood pressure.^2^ Lastly, a decline in oestrogen may lead to increases in proinflammatory cytokines and therefore vascular inflammation, elevating the likelihood of CVD.^2 21^ All these pathways may accelerate the progression of atherosclerosis, and consequently, those with earlier menopause are exposed to this CVD earlier in life.^22^ This reiterates the importance of early lifestyle interventions to offset the likelihood of CVD during and after menopause.

Lifestyle modification is a key aspect of CVD prevention and reducing CVD burden.^24^ Unhealthy lifestyle behaviours are well-established risk factors in postmenopausal and midlife women.^2 9^ Those adhering to healthier lifestyles, including a healthy diet, exercise, and not smoking, significantly mitigate their probability of developing future CVD.^2 11^ Few studies have prospectively examined the combined effects of lifestyle factors on CVD in postmenopausal and midlife women.^10 25^ In 2018, the Study of Women’s Health Across the Nation (SWAN) found that the Healthy-lifestyle Score (HLS), composed of three modifiable CVD risk factors (smoking, diet and physical activity), was associated with less subclinical atherosclerosis.^25^ Most recently, in 2023, the Healthy-lifestyle Index (HLI) was assessed in a large prospective cohort from the Women’s Health Initiative of >40 000 participants, concluding that postmenopausal women who had a healthier lifestyle significantly lowered their CVD risk.^10^ These studies have been largely built on the American Heart Association’s Life’s Simple 7 goals of smoking, physical activity, BMI, blood pressure, blood glucose and total cholesterol.^25^ Additionally, these studies suggest that the combined effect of overall lifestyle is more effective in reducing the likelihood of CVD rather than individual lifestyle behaviours.^10 11^

Our combined healthy lifestyle score differs from previous scores by quantifying a unique combination of lifestyle factors more relevant in middle-aged and older Australian women, such as sitting and sleep in addition to smoking, physical activity and diet.^15^ We found that both high and medium adherence to this healthy lifestyle significantly attenuated the likelihood of CVD by 23% and 15%, respectively, in women who have reached menopause. This remained significant after additional adjustment for alcohol intake. Our findings were consistent with Peila *et al*’s^10^ study that found a healthier lifestyle, assessed by the highest HLI, led to a 26% reduction in of the probability of CVD. This study was most comparable to our analysis and menopausal cohort, with similar follow-up duration (15-year vs 20-year follow-up), sample size (N=46 238 vs 40 118), CVD event rate (11.7% vs 9.5%) and mean age (62.1 years vs 63 years).^10^ Two meta-analyses have indicated that higher lifestyle indices were associated with greater reductions in CVD by 62%–66%.^24 26^ However, this study was limited due to the lack of sex-disaggregated analyses and did not include sitting and sleep duration, both of which have been linked to all-cause mortality and CVD.^16 17^

Evidence on the primary prevention of CVD with healthier lifestyles in middle-aged and older women is strong and consistent. However, both randomised and observational studies have not adequately tested the benefits of healthier lifestyles in high-risk female populations, particularly those of earlier menopause.^2^ Our study prospectively examined the interaction effects between age of menopause and lifestyle on the likelihood of CVD. We found that this interaction did not reach statistical significance, suggesting that combined impact of healthy lifestyle was not significantly different across the menopause categories on the likelihood of CVD, despite the downward trend in the ORs. While we did find a significant effect of a healthy lifestyle in women with premature menopause and women with menopause at mean age of 50–52 years, absolute conclusions cannot be made. Our results may have been impacted by a sample size that was not large enough to have adequate statistical power for interaction analyses. Nevertheless, this emphasises the necessity to design more robust studies to explore healthy lifestyles in women with premature/early menopause and whether tailored advice is needed specifically for these women.

### Limitations

First, the study was observational in nature, and missing data were present, therefore there may be unmeasured confounders that could affect the outcome. Multiple imputations were performed for missing data in sensitivity analyses to limit this bias. Second, our study was limited by using only self-reported outcomes and was subject to loss to follow-up, for example, we were unable to determine the proportion of fatal CVD, potentially leading to outcome misclassification. However, the validity of self-reported responses is important to consider and has been well reported in the literature, for example, an Australian study previously found only 0.2% of CVD events were undisclosed in self-reported studies.^27^ Additionally, for female-specific risk factors (HDP and GDM), prior studies^28 29^ have reported good concordance between actual reports and self-reported responses, while the validity of some cardiovascular risk factors (eg, hypertension, DM and smoking) can be variable.^30^ Furthermore, there may be potential selection bias due to loss to follow-up and excluding those with missing outcome data (38%). We acknowledge there were significant differences in baseline characteristics between those who had information on CVD outcome and those who did not may have led to attrition bias. However, our CVD event rate (11.7% vs 9.5%) was similar to Peila *et al*’s^10^ cohort of >40 000 postmenopausal women that used validated hospital records to ascertain CVD outcome, with comparable baseline age (average 62.07 years vs 63 years) and follow-up (15 year vs 20 year). Third, lifestyle factors were self-reported and assessed using the baseline questionnaire. Lifestyle behaviours were measured only at baseline in the current study. We acknowledge that lifestyle behaviours can change over time, and changes in lifestyle behaviours may affect the likelihood of CVD. Moreover, the diet component of our score was measured using a general dietary assessment and not a food-frequency questionnaire, limiting our ability to test a more comprehensive diet score. Lastly, our sample size may have been underpowered to detect a significant interaction effect. Thus, the interaction effects across menopause categories should be interpreted with caution. It should be noted that while the baseline response rate in the 45 and Up Study was modest (~19%), representativeness is not important in cohort studies; observed cross-sectional exposure-outcome relationships were similar to state-based surveillance systems reporting higher response rates.^13 31^

### Future directions

We have identified that women with premature/early menopause are at higher odds of CVD events and that a healthy lifestyle can mitigate this likelihood. Future research should therefore focus on interventions that can improve healthy lifestyles and that assess the impact of this on long-term CVD. There is an unmet need to implement preventative interventions and management of cardiovascular risk factors in high-risk women with premature/early menopause.

## Conclusion

Women with premature and early menopause, before the age of 40 and 45 years, respectively, are at an increased likelihood of CVD. This increased likelihood of CVD is significantly reduced in all women adhering to a healthy lifestyle. The menopause change offers an ideal time to screen women for cardiovascular risk factors and intervene with healthy lifestyle advice.

## supplementary material

10.1136/heartjnl-2024-324602 online supplemental file 1

## Data Availability

No data are available.
