# Supplementary material for: Age of menopause, healthy lifestyle and cardiovascular disease in women: a prospective cohort study
Source: Heart. 2024 Dec 17;111(6):e324602. doi: 10.1136/heartjnl-2024-324602 (PMC11874333; doi:10.1136/heartjnl-2024-324602)
Supplement: online supplemental file 1 [file heartjnl-111-6-s001.pdf]

Supplemental material

Table of Contents

Table S1. Questions and response options used in the 45 and Up Study baseline questionnaire to assess lifestyle risk factors 2

Table S2. Questions and response options for variables in the 45 and Up Study baseline questionnaire 3

Figure S1. Study flow chart participants following the inclusion/exclusion criteria. Women aged ≥ 45 years with a 15-year follow-up in the Sax Institute's 45 and Up Study Cohort were included. *CVD*, cardiovascular disease. 6

Table S3. Baseline characteristics according to menopausal categories in women from the 45 and Up Cohort (n=46,238). 7

Table S4. Sensitivity analyses adjusting for female-specific variables on the association between cardiovascular disease and menopause categories in women from the 45 and Up Cohort (n=46,238). 11

Table S5. Sensitivity analyses adjusting for lifestyle behaviours on the association between cardiovascular disease and menopause categories in women from the 45 and Up Cohort (n=46,238). 12

Table S6. OR for the association between cardiovascular disease and healthy-lifestyle adherence by menopause categories in women from the 45 and Up Cohort (n=41,672). 13

Table S7. Sensitivity analysis after imputing for missing values for the association between cardiovascular disease and menopause categories in women from the 45 and Up Cohort (n=46,238). 14

Table S8. Sensitivity analysis after imputing for missing values for the association between cardiovascular disease and overall lifestyle adherence in women from the 45 and Up Cohort (n=46,238). 15

Table S9. Sensitivity analysis after imputing for missing values for the association between cardiovascular disease and healthy-lifestyle adherence by menopause categories in from the 45 and Up Cohort (n=46,238). 16

Table S1. Questions and response options used in the 45 and Up Study baseline questionnaire to assess lifestyle risk factors

Lifestyle	Question	Response options	Derivation
**Smoking**	Are you a regular smoker now?<br>Have you ever been a regular smoker?	Yes/No<br>Yes/No	Categorised as: Non-smoker, Previous smoker, Current smoker
**Physical activity**	How many TIMES did you do each of these activities LAST WEEK?<br>-Walking continuously, for at least 10 minutes (for recreation or exercise or to get to or from places)<br>-Vigorous physical activity (that made you breathe harder or puff and pant, like jogging, cycling, aerobics, competitive tennis, but not household chores or gardening)<br>-Moderate physical activity (like gentle swimming, social tennis, vigorous gardening or work around the house)	__times in the last week (for walking, vigorous physical activity and moderate physical activity)	
	If you add up all the time you spent doing each activity LAST WEEK, how much time did you spend ALTOGETHER doing each type of activity?	__hours:__minutes (for walking, vigorous physical activity and moderate physical activity)	Categorised as: ≥300, 150–299, ≤149
**Sleep**	About how many HOURS in each 24 hour DAY do you usually spend doing the following? (please put “0” if you do not spend any time doing it)	Hours per day __sleeping (including at night & naps)	Categorised as: 7–9, >5–7 or >9–11, <5 or >11
**Sitting**	About how many HOURS in each 24 hour DAY do you usually spend doing the following? (please put “0” if you do not spend any time doing it)	Hours per day __sitting	Categorised as: <7, 7–9, >9
**Diet score**			
**Fruit**	About how many serves of fruit do you usually have each day? A serve is 1 medium piece or 2 small pieces or 1 cup diced or canned fruit pieces	__number of serves of fruit each day	Categorised as: ≥2, 1, 0
**Vegetable**	About how many serves of vegetables do you usually eat each day? A serve is half a cup of cooked vegetables or one cup of salad (please include potatoes and put “0” is less than one a day)	__number of serves of cooked vegetables each day<br>__number of serves of raw vegetables each day (e.g. salad)	Categorised as: ≥5, 3–4, 0–2
**Red meat**	About how many times each week do you eat “beef, lamb or pork”	__number of times eaten each week	Categorised as: 0–2, 3–4, ≥5
**Processed meat**	About how many times each week do you eat “processed meat (include bacon, sausages, salami, devon, burgers,etc)”	__number of times eaten each week	Categorised as: 0, 1–2, ≥3
**Fish**	About how many times each week do you eat “fish or seafood”	__number of times eaten each week	Categorised as: ≥3, 1–2, 0

Table S2. Questions and response options for variables in the 45 and Up Study baseline questionnaire

Variable	Question	Response options	Derivation
**Age of menopause onset (continuous)**	How old were you when you had your menopause?	Age in years	
**BMI (continuous)**	Derived from self-reported height (About how tall are you?) and weight (About how much do you weigh?).	__meters<br><br>__kilograms	Weight in Kilograms / (Height in Meters x Height in Meters)
**BMI group (categorical)**	Assessed using the BMI data item (above)		Categorised as:<br>Underweight <18.5<br>Healthy weight 18.5–24.99<br>Overweight 25.0–29.99<br>Obesity ≥ 30
**Ancestry (categorical)**	What is your ancestry? (Yes/No)	Australian<br>English<br>Irish<br>Chinese<br>Italian<br>Greek<br>Scottish<br>German<br>Lebanese<br>Dutch<br>Maltese<br>Polish<br>Filipino<br>Indian<br>Croatian<br>Vietnamese<br>Other	Categorised as:<br>Australian<br><br>European (English or Irish or Italian or Greek or Scottish or Dutch or Maltese or Polish or Croatian)<br><br>Asian (Chinese or Lebanese or Filipino or Indian or Vietnamese)<br><br>Other
**Highest qualification (categorical)**	What is the highest qualification you have completed? (Yes/No)	1 = No school certificate or other qualification<br>2 = School or intermediate certificate<br>3 = Higher school or leaving certificate<br>4 = Trade or apprenticeship<br>5 = Certificate or diploma<br>6 = University degree or higher	

Table S2. Continued.

Variable	Question	Response options	Derivation
**Marital status (categorical)**	What best describes your current situation? (Yes/No)	1 = Single<br>2 = Married<br>3 = De facto/living with partner<br>4 = Widowed<br>5 = Divorced<br>6 = Separated	Categorised as:<br>Married/Defacto (2 or 3)<br>Divorced/Separated (5 or 6)<br>Widowed/Single (1 or 4)
**Remoteness (categorical)**	Based on information from the 2006 Accessibility and Remoteness Index of Australia Plus score of participants' residential postcode	1 = Major Cities of Australia<br>2 = Inner Regional Australia<br>3 = Outer Regional Australia<br>4 = Remote Australia<br>5 = Very Remote Australia	Categorised as:<br>Metropolitan (1)<br>Inner Regional (2)<br>Outer regional/Remote/Very Remote (3 or 4 or 5)
**Household annual income ($AUD) (categorical)**	What is your usual yearly household income before tax from all sources? Per year (Yes/No)	1 = less then $5,000 per year<br>2 = $5,000-$9,999 per year<br>3 = $10,000-$19,999 per year<br>4 = $20,000-$29,999 per year<br>5 = $30,000-$39,999 per year<br>6 = $40,000-$49,999 per year<br>7 = $50,000-$69,999 per year<br>8 = $70,000 or more per year	Categorised as:<br><$30,000 (1 or 2 or 3 or 4)<br>$30,000-$69,999 (5 or 6 or 7)<br>>$70,000 (8)
**Parity (continuous)**	How many children have you given birth to?	__number of children given birth	
**Parity (categorical)**	Assessed using the data item for parity (above)		Categorised as 0 children, 1-2 children, and 3+ children
**History of HDP (categorical)**	Has a doctor ever told you that you have high blood pressure when pregnant	Yes/No	
**Hysterectomy (categorical)**	Have you had any of the following operations? hysterectomy	Yes/No	
**History of OCP (categorical)**	Have you ever taken the oral contraceptive pill	Yes/No	

Table S2. Continued.

Variable	Question	Response options	Derivation
**History of Menopausal hormonal therapy (categorical)**	Have you ever used hormone replacement therapy?	Yes/No	
**DM (categorical)**	Has a doctor ever told you that you have Diabetes	Yes/No	
**Hypertension (categorical)**	Has a doctor ever told you that you have: High Blood Pressure when NOT pregnant	Yes/No	
**Age (continuous)**	Derived from date of birth and year	Age in years	
**Gender (categorical)**	What is your gender	Male/Female	

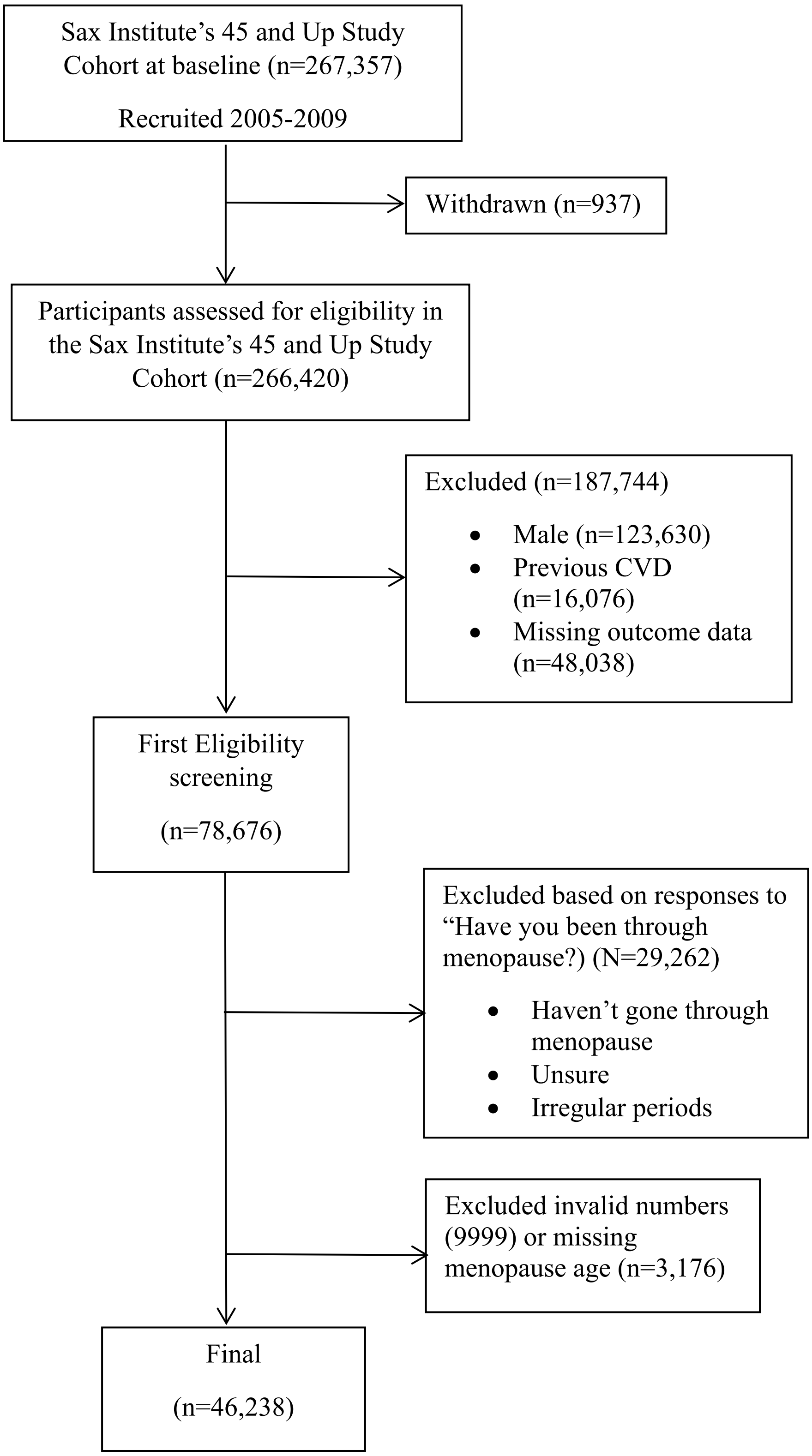

Figure S1. Study flow chart participants following the inclusion/exclusion criteria. Women aged ≥ 45 years with a 15-year follow-up in the Sax Institute's 45 and Up Study Cohort were included. *CVD*, cardiovascular disease.

Table S3. Baseline characteristics according to menopausal categories in women from the 45 and Up Cohort (n=46,238).

Characteristics	Premature menopause (<40 years)	Early menopause (40-44 years)	Relatively early menopause (45-49 years)	Menopause at 50-52 years	Late menopause (>52 years)	Total (n=46,238)
**Number of participants (%)**	1,704 (3.68%)	4,297 (9.29%)	12,026 (26.00%)	16,643 (35.99%)	11,578 (25.04%)	46,238
**Baseline age (mean, SD years)**	61.39±9.49	61.09±9.07	60.84±8.77	62.28±8.04	63.53±7.13	62.07±8.24
**Age at menopause onset (mean, SD years)**	34.83±4.54	41.86±1.47	47.04±1.50	50.72±0.87	55.04±2.08	49.44±5.10
**BMI (mean, SD)**	27.70±5.84	26.89±5.34	26.44±5.11	26.32±4.98	26.53±4.98	26.51±5.09
**BMI groups (kg/m^2) (%)**						
**Underweight <18.5**	165 (9.68%)	405 (9.43%)	974 (8.11%)	1,412 (8.48%)	950 (8.21%)	3,907 (8.45%)
**Healthy weight 18.5–24.99**	567 (33.27%)	1,556 (36.21%)	4,906 (40.83%)	6,926 (41.62%)	4,568 (39.45%)	18,542 (40.06%)
**Overweight 25.0–29.99**	491 (28.81%)	1,375 (32.00%)	3,799 (31.62%)	5,238 (31.47%)	3,761 (32.48%)	14,664 (31.71%)
**Obesity ≥ 30**	481 (28.23%)	961 (22.36%)	2,337 (19.45%)	3,067 (18.43%)	2,299 (19.86%)	9,145 (19.78%)
**Ancestry (%)**						
**Australian**	370 (22.05%)	927 (21.81%)	2,559 (21.48%)	3,481 (21.12%)	2,406 (20.94%)	9,747 (21.27%)
**European**	1,121 (66.81%)	2,824 (66.45%)	7,928 (66.56%)	11,037 (66.97%)	7,749 (67.45%)	30,675 (66.93%)
**Asian**	33 (1.97%)	84 (1.98%)	300 (2.52%)	352 (2.14%)	213 (1.85%)	982 (2.14%)
**Other**	154 (9.18%)	415 (9.76%)	1,124 (9.44%)	1,611 (9.77%)	1,120 (9.75%)	4,428 (9.66%)
Missing						*441 (0.95%)*
**Highest qualification (%)**						
**No school certificate or other qualification**	239 (14.20%)	499 (11.73%)	1,103 (9.27%)	1,304 (7.90%)	835 (7.27%)	3,980 (8.68%)
**School or intermediate certificate**	536 (31.85%)	1,251 (29.40%)	3,238 (27.20%)	4,364 (26.44%)	3,112 (27.08%)	12,501 (27.27%)
**Higher school or leaving certificate**	153 (9.09%)	389 (9.14%)	1,128 (9.48%)	1,563 (9.47%)	1,038 (9.03%)	4,271 (9.32%)
**Trade or apprenticeship**	102 (6.06%)	200 (4.70%)	505 (4.24%)	620 (3.76%)	432 (3.76%)	1,859 (4.06%)
**Certificate or diploma**	390 (23.17%)	982 (23.08%)	2,923 (24.55%)	4,069 (24.65%)	2,748 (23.91%)	11,112 (24.24%)
**University degree or higher**	263 (15.63%)	934 (21.95%)	3,008 (25.27%)	4,586 (27.78%)	3,327 (28.95%)	12,118 (26.43%)
Missing						*397 (0.86%)*

**Table S3.** (continued)						
**Characteristics**	**Premature menopause (<40 years)**	**Early menopause (40-44 years)**	**Relatively early menopause (45-49 years)**	**Menopause at 50-52 years**	**Late menopause (>52 years)**	**Total (n=46,238)**
**ARIA+ group (%)**						
**Metropolitan**	803 (48.08%)	2,066 (49.18%)	6,011 (50.95%)	8,356 (51.15%)	5,556 (49.02%)	22,792 (50.27%)
**Inner regional**	634 (37.96%)	1,652 (39.32%)	4,383 (37.15%)	6,138 (37.57%)	4,413 (38.94%)	17,220 (37.98%)
**Outer regional/Remote/Very Remote**	233 (13.95%)	483 (11.50%)	1,403 (11.89%)	1,842 (11.89%)	1,365 (12.04%)	5,326 (11.75%)
Missing						*900 (1.94%)*
**Marital status (%)**						
**Married/Defacto**	1,168 (68.79%)	3,016 (70.34%)	8,672 (72.33%)	12,228 (73.65%)	8,674 (75.07%)	33,758 (73.17%)
**Divorced/Separated**	216 (12.72%)	564 (13.15%)	1,487 (12.40%)	1,922 (11.58%)	1,314 (11.37%)	5,503 (11.93%)
**Widowed/Single**	314 (18.50%)	708 (16.51%)	1,831 (15.27%)	2,453 (14.77%)	1,567 (13.56%)	6,873 (14.89%)
Missing						*104 (0.22%)*
**Household annual income ($AUD) (%)**						
**<$30,000**	605 (37.05%)	1,344 (32.24%)	3,300 (28.19%)	4,371 (26.96%)	3,286 (29.14%)	12,905 (28.68%)
**$30,000-$69,999**	439 (26.88%)	1,104 (26.48%)	3,383 (28.90%)	4,792 (29.58%)	3,301 (29.28%)	13,020 (28.94%)
**>$70,000**	252 (15.43%)	866 (20.77%)	2,745 (23.45%)	3,826 (23.61%)	2,481 (22.00%)	10,170 (22.61%)
Prefer not to answer						*8,808 (19.58%)*
Missing						*1,338 (2.89%)*
**Parity (no. of children given birth) (%)**						
**0**	281 (16.55%)	571 (13.33%)	1,635 (13.67%)	1,856 (11.19%)	1,013 (8.78%)	5,356 (11.63%)
**1-2**	727 (42.81%)	1,928 (45.02%)	5,326 (44.53%)	7,266 (43.80%)	4,957 (42.98%)	20,204 (43.86%)
**3+**	690 (40.64%)	1,784 (41.65%)	4,999 (41.80%)	7,470 (45.02%)	5,563 (48.24%)	20,506 (44.51%)
Missing						*172 (0.37%)*
**History of HDP (%)**	248 (4.55%)	462 (8.47%)	1,317 (25.14%)	1,919 (35.19%)	1,454 (26.99%)	5,454 (11.80%)
**Hysterectomy (%)**	903 (52.99%)	1,328 (30.91%)	2,603 (21.66%)	2,839 (17.06%)	1,938 (16.74%)	9,611 (20.79%)
**History of OCP (%)**	1,361 (80.96%)	3,558 (84.09%)	9,956 (83.87%)	13,705 (83.35%)	9,666 (84.60%)	38,246 (83.78%)
Missing						*590 (1.28%)*
**History of MHT (%)**	370 (21.71%)	613 (14.27%)	1,437 (11.96%)	1,560 (9.37%)	868 (7.50%)	4,849 (10.48%)
Missing						*15,563 (33.7%)*
**DM(%)**	153 (8.98%)	242 (5.63%)	595 (4.95%)	803 (4.82%)	592 (5.11%)	2,385 (5.16%)

**Table S3.** (continued)						
**Characteristics**	**Premature menopause (<40 years)**	**Early menopause (40-44 years)**	**Relatively early menopause (45-49 years)**	**Menopause at 50-52 years**	**Late menopause (>52 years)**	**Total (n=46,238)**
**Hypertension (%)**	643 (37.73%)	1,338 (31.14%)	3,573 (29.74%)	5,346 (32.12%)	4,059 (35.06%)	14,959 (32.35%)
**Smoking status (%)**						
**Current smoker**	200 (11.74%)	359 (8.35%)	843 (7.02%)	674 (4.05%)	323 (2.79%)	2,399 (5.19%)
**Previous smoker**	539 (31.63%)	1,383 (32.19%)	3,681 (30.63%)	4,649 (27.94%)	3,043 (26.28%)	13,295 (28.75%)
**Non-smoker**	965 (56.63%)	2,555 (59.46%)	7,492 (62.35%)	11,319 (68.01%)	8,212 (70.93%)	30,543 (66.06%)
Missing						*1 (0.002%)*
**Physical activity (Minutes of MVPA per week) (%)**						
**≤149**	470 (30.44%)	1,126 (28.51%)	3,133 (27.78%)	4,218 (27.00%)	2,784 (25.63%)	11,731 (27.12%)
**150–299**	251 (16.26%)	673 (17.04%)	2,011 (17.83%)	2,750 (17.60%)	1,946 (17.92%)	7,631 (17.64%)
**≥300**	823 (53.30%)	2,150 (54.44%)	6,133 (54.39%)	8,653 (55.39%)	6,131 (56.45%)	23,890 (55.23%)
Missing						*2,986 (6.50%)*
**Sitting (hours per day) (%)**						
**>9**	162 (10.18%)	437 (10.76%)	1,306 (11.42%)	1,773 (11.24%)	1,170 (10.65%)	4,848 (11.06%)
**7-9**	268 (16.84%)	688 (16.93%)	1,987 (17.37%)	2,661 (16.87%)	1,744 (15.88%)	7,248 (16.76%)
**<7**	1,161 (72.97%)	2,939 (72.31%)	8,146 (71.21%)	11,342 (71.89%)	8,067 (73.45%)	31654 (72.19%)
Missing						*2,388 (5.16%)*
**Sleep (hours per day) (%)**						
**<5 or >11**	57 (3.40%)	125 (2.95%)	256 (2.15%)	288 (1.75%)	237 (2.07%)	963 (2.11%)
**>5–7 or >9–11**	410 (24.48%)	936 (22.06%)	2,230 (18.75%)	2,863 (17.38%)	1,970 (17.20%)	8,409 (18.39%)
**7–9**	1,208 (72.12%)	3,182 (74.99%)	9,406 (79.10%)	13,323 (80.87%)	9,245 (80.73%)	36,364 (79.51%)
Missing						*502 (1.09%)*
**Diet score (out of 10) (%)**						
**0-3**	92 (5.42%)	237 (5.53%)	448 (4.65%)	690 (4.15%)	435 (3.77%)	2,012 (4.36%)
**>3-7**	866 (51.03%)	2,191 (51.16%)	6,025 (50.25%)	8,019 (48.28%)	5,440 (47.09%)	22,541 (48.86%)
**<7**	739 (43.55%)	1,855 (43.31%)	5,406 (45.09%)	7,900 (47.56%)	5,678 (49.15%)	21,578 (46.78%)
Missing						*107 (0.23%)*

**Table S3.** (continued)						
**Characteristics**	**Premature menopause (<40 years)**	**Early menopause (40-44 years)**	**Relatively early menopause (45-49 years)**	**Menopause at 50-52 years**	**Late menopause (>52 years)**	**Total (n=46,238)**
**Overall healthy-lifestyle score (out of 10) (%)**						
**0-5**	227 (13.72%)	506 (12.02%)	1,261 (10.64%)	1,370 (8.36%)	858 (7.53%)	4,222 (9.28%)
**6-8**	1,010 (61.03%)	2,485 (59.04%)	6,962 (58.72%)	9,409 (57.45%)	6,373 (55.94%)	26,233 (57.68%)
**9-10**	418 (25.26%)	1,218 (28.94%)	3,630 (30.64%)	5,599 (34.19%)	4,161 (36.53%)	15,026 (33.04%)
Missing						*757 (1.64%)*

BMI, body mass index; *ARIA+*, Accessibility/Remoteness Index of Australia Plus; *AUD,* Australian dollars; *HDP*, hypertension during pregnancy; *OCP,* oral contraceptive pill; *MHT,* menopausal hormone therapy; *DM*, diabetes mellitus; *MVPA*, moderate to vigorous physical activity.
[1] *The full baseline survey is available at https://www.saxinstitute.org.au/our-work/45-up-study/questionnaires/*

Table S4. Sensitivity analyses adjusting for female-specific variables on the association between cardiovascular disease and menopause categories in women from the 45 and Up Cohort (n=46,238).

	**Menopause at 50-52 years**	**Premature menopause (<40 years)**		**Early menopause (40-44 years)**		**Relatively early menopause (45-49 years)**		**Late menopause (>52 years)**	
	Reference	OR	95% CI, p value	OR	95% CI, p value	OR	95% CI, p value	OR	95% CI, p value
**Adjustments** [a]									
Additional adjustment for HDP	1.0 (Reference)	1.35	(1.17-1.56), **p<0.0001**	1.15	(1.04-1.28), **p=0.008**	1.07	(0.99-1.16), p=0.07	1.00	(0.92-1.07), p=0.91
Additional adjustment for hysterectomy	1.0 (Reference)	1.30	(1.13-1.51), **p<0.001**	1.13	(1.02-1.26), **p=0.021**	1.07	(0.99-1.15), p=0.10	1.00	(0.93-1.08), p=0.95
Additional adjustment for OCP	1.0 (Reference)	1.36	(1.18-1.57), **p<0.0001**	1.15	(1.04-1.28), **p=0.008**	1.07	(0.99-1.16), p=0.07	1.00	(0.92-1.07), p=0.91
Additional adjustment for parity	1.0 (Reference)	1.35	(1.17-1.56), **p<0.0001**	1.14	(1.03-1.27), **p=0.012**	1.07	(0.99-1.16), p=0.07	0.99	(0.92-1.07), p=0.82
Additional adjustment for hysterectomy, OCP, parity, and HDP	1.0 (Reference)	1.30	(1.12-1.50), **p<0.001**	1.13	(1.02-1.25), **p=0.024**	1.07	(0.99-1.15), p=0.10	0.99	(0.92-1.07), p=0.81

[a] Adjusted for: age, body mass index (BMI), ancestry, baseline hypertension (HTN), baseline diabetes mellitus (DM), highest qualification, smoking status, and menopausal hormone therapy (MHT).

CI, confidence intervals; *HDP,* hypertension during pregnancy; *OCP,* oral contraceptive pill; *OR*, odds ratio.

All ORs and p values are in reference to mean age of menopause at 50-52 years

Bolded p-values are considered statistically significant at p<0.05.

Table S5. Sensitivity analyses adjusting for lifestyle behaviours on the association between cardiovascular disease and menopause categories in women from the 45 and Up Cohort (n=46,238).

	**Menopause at 50-52 years**	**Premature menopause (<40 years)**		**Early menopause (40-44 years)**		**Relatively early menopause (45-49 years)**		**Late menopause (>52 years)**	
	Reference	OR	95% CI, p value	OR	95% CI, p value	OR	95% CI, p value	OR	95% CI, p value
**Adjustments** [a]									
Additional adjustment for physical activity	1.0 (Reference)	1.38	(1.19-1.59), **p<0.001**	1.16	(1.05-1.29), **p=0.005**	1.08	(1.00-1.16), p=0.051	0.99	(0.92-1.07), p=0.89
Additional adjustment for sleep duration	1.0 (Reference)	1.35	(1.16-1.57), **p<0.0001**	1.15	(1.04-1.28), **p=0.009**	1.07	(1.00-1.16), p=0.06	0.99	(0.91-1.06), p=0.70
Additional adjustment for sitting time	1.0 (Reference)	1.37	(1.31-1.44), **p<0.0001**	1.17	(1.13-1.21), **p<0.001**	1.08	(1.06-1.11), **p<0.001**	0.99	(0.96-1.01), p=0.26
Additional adjustment for diet quality	1.0 (Reference)	1.37	(1.18-1.58), **p<0.0001**	1.17	(1.05-1.30), **p=0.005**	1.08	(1.00-1.17), **p=0.04**	0.99	(0.91-1.06), p=0.69
Additional adjustment for overall healthy lifestyle adherence	1.0 (Reference)	1.35	(1.17-1.56), **p<0.001**	1.15	(1.04-1.28), **p=0.008**	1.08	(1.00-1.16), p=0.06	0.99	(0.92-1.07), p=0.77

[a] Adjusted for: age, body mass index (BMI), ancestry, baseline hypertension (HTN), baseline diabetes mellitus (DM), highest qualification, smoking status, and menopausal hormone therapy (MHT).
CI, confidence intervals; *OR*, odds ratio.
All ORs and p values are in reference to mean age of menopause at 50-52 years
Bolded p-values are considered statistically significant at p<0.05.

Table S6. OR for the association between cardiovascular disease and healthy-lifestyle adherence by menopause categories in women from the 45 and Up Cohort (n=41,672).

	Low healthy-lifestyle adherence	Medium healthy-lifestyle adherence		High health-lifestyle adherence	
	Reference	OR	95% CI, p value	OR	95% CI, p value
**Premature menopause (<40 years)**					
Unadjusted	1.0 (Reference)	0.70	(0.49-1.01), p=0.054	0.51	(0.33-0.79), **p=0.003**
Adjusted*	1.0 (Reference)	0.65	(0.43-0.96), **p=0.030**	0.48	(0.30-0.77), **p=0.002**
**Early menopause (40-44 years)**					
Unadjusted	1.0 (Reference)	0.95	(0.71-1.26), p=0.71	0.90	(0.66-1.23), p=0.50
Adjusted*	1.0 (Reference)	0.93	(0.68-1.27), p=0.64	0.85	(0.61-1.20), p=0.36
**Relative early menopause (45-49 years)**					
Unadjusted	1.0 (Reference)	0.95	(0.79-1.14), p=0.54	0.87	(0.71-1.06), p=0.16
Adjusted*	1.0 (Reference)	0.88	(0.72-1.08), p=0.23	0.83	(0.67-1.03), p=0.08
**Menopause at 50-52 years**					
Unadjusted	1.0 (Reference)	0.83	(0.70-0.98), **p=0.024**	0.74	(0.62-0.89), **p=0.001**
Adjusted*	1.0 (Reference)	0.82	(0.69-0.99), **p=0.038**	0.77	(0.63-0.93), **p=0.0068**
**Late menopause (>52 years)**					
Unadjusted	1.0 (Reference)	0.91	(0.73-1.12), p=0.35	0.78	(0.63-0.97), **p=0.028**
Adjusted*	1.0 (Reference)	0.90	(0.72-1.13), p=0.37	0.79	(0.63-1.01), p=0.06

*Adjusted for: age, body mass index (BMI), ancestry, baseline hypertension (HTN), baseline diabetes mellitus (DM), highest qualification, remoteness, and menopausal hormone therapy (MHT).

CI, confidence intervals; *CVD*, cardiovascular disease; *OR*, odds ratio. Bolded p-values are considered statistically significant at p<0.05

Table S7. Sensitivity analysis after imputing for missing values for the association between cardiovascular disease and menopause categories in women from the 45 and Up Cohort (n=46,238).

	**Menopause at 50-52 years**	**Premature menopause (<40 years)**		**Early menopause (40-44 years)**		**Relatively early menopause (45-49 years)**		**Late menopause (>52 years)**	
	Reference	OR	95% CI, p value	OR	95% CI, p value	OR	95% CI, p value	OR	95% CI, p value
**CVD**									
Unadjusted	1.0 (Reference)	1.43	(1.25-1.65), **p<0.0001**	1.13	(1.02-1.25), **p=0.017**	1.02	(0.94-1.09), p=0.68	1.06	(0.98-1.14), p=0.15
Adjusted*	1.0 (Reference)	1.36	(1.17-1.57), **p<0.0001**	1.15	(1.04-.1.28), **p= 0.009**	1.07	(0.99-1.16), p=0.07	1.00	(0.92-1.07), p=0.93

*Adjusted for: age, body mass index (BMI), ancestry, baseline hypertension (HTN), baseline diabetes mellitus (DM), highest qualification, remoteness, and menopausal hormone therapy (MHT).
CI, confidence intervals; *CVD*, cardiovascular disease; *OR*, odds ratio.
All ORs and p values are in reference to mean age of menopause at 50-52 years.
Bolded p-values are considered statistically significant at p<0.05.

Table S8. Sensitivity analysis after imputing for missing values for the association between cardiovascular disease and overall lifestyle adherence in women from the 45 and Up Cohort (n=46,238).

	**Low healthy-lifestyle adherence**	**Medium healthy-lifestyle adherence**		**High health-lifestyle adherence**	
	Reference	OR	95% CI, p value	OR	95% CI, p value
**CVD**					
Unadjusted	1.0 (Reference)	0.87	(0.79-0.96), **p=0.005**	0.78	(0.70-0.86), **p<0.0001**
Adjusted*	1.0 (Reference)	0.84	(0.76-0.92), **p<0.001**	0.75	(0.68-0.83), **p<0.0001**
Additional adjustment for weekly alcohol intake	1.0 (Reference)	0.83	(0.76-0.92), **p<0.001**	0.74	(0.66-0.82), **p<0.0001**

*Adjusted for: age, body mass index (BMI), ancestry, baseline hypertension (HTN), baseline diabetes mellitus (DM), highest qualification, remoteness, and menopausal hormone therapy (MHT).

CI, confidence intervals; *CVD*, cardiovascular disease; *OR*, odds ratio. Bolded p-values are considered statistically significant at p<0.05

Table S9. Sensitivity analysis after imputing for missing values for the association between cardiovascular disease and healthy-lifestyle adherence by menopause categories in from the 45 and Up Cohort (n=46,238).

	**Low healthy-lifestyle adherence**	**Medium healthy-lifestyle adherence**		**High health-lifestyle adherence**	
	Reference	OR	95% CI, p value	OR	95% CI, p value
**Premature menopause (<40 years)**					
Unadjusted	1.0 (Reference)	0.70	(0.49-1.01), p=0.054	0.51	(0.33-0.79), **p=0.003**
Adjusted*	1.0 (Reference)	0.65	(0.43-0.96), **p=0.030**	0.48	(0.30-0.75), **p=0.001**
**Early menopause (40-44 years)**					
Unadjusted	1.0 (Reference)	0.95	(0.71-1.26), p=0.71	0.90	(0.66-1.23), p=0.50
Adjusted*	1.0 (Reference)	0.91	(0.68-1.21), p=0.51	0.84	(0.62-1.16), p=0.29
**Relative early menopause (45-49 years)**					
Unadjusted	1.0 (Reference)	0.95	(0.79-1.14), p=0.54	0.87	(0.71-1.06), p=0.16
Adjusted*	1.0 (Reference)	0.87	(0.72-1.05), p=0.15	0.79	(0.65-0.97), **p=0.026**
**Menopause at 50-52 years**					
Unadjusted	1.0 (Reference)	0.83	(0.70-0.98), **p=0.024**	0.74	(0.62-0.89), **p=0.001**
Adjusted*	1.0 (Reference)	0.80	(0.67-0.95), **p=0.012**	0.74	(0.61-0.88), **p=0.0009**
**Late menopause (>52 years)**					
Unadjusted	1.0 (Reference)	0.91	(0.73-1.12), p=0.35	0.78	(0.63-0.97), **p=0.028**
Adjusted*	1.0 (Reference)	0.92	(0.74-1.13), p=0.42	0.81	(0.65-1.01), p=0.06

*Adjusted for: age, body mass index (BMI), ancestry, baseline hypertension (HTN), baseline diabetes mellitus (DM), highest qualification, remoteness, and menopausal hormone therapy (MHT).

CI, confidence intervals; *CVD*, cardiovascular disease; *OR*, odds ratio. Bolded p-values are considered statistically significant at $p<0.05$
